# Rice bran nanofiber composites for stabilization of phytase

**DOI:** 10.1186/s13065-018-0400-y

**Published:** 2018-03-14

**Authors:** Upendra A. Rathnayake, Tharindu Senapathi, Chanaka Sandaruwan, Sanja Gunawardene, Veranja Karunaratne, Nilwala Kottegoda

**Affiliations:** 1 Sri Lanka Institute of Nanotechnology, Centre for Excellence, Nanoscience and Technology Park, Pitipana, Homagama, Sri Lanka; 2 Department of Chemical and Process Engineering, University of Moratuwa, Moratuwa, Sri Lanka; 3 Department of Chemistry, University of Sri Jayewardenepura, Nugegoda, Sri Lanka; 4 Center for Advanced Material Research, University of Sri Jayewardenepura, Nugegoda, Sri Lanka

**Keywords:** Nanofibers, Encapsulation, Phytase enzyme, Electrospinning, Thermal stability

## Abstract

This study explores the potential application of rice bran (agro waste) to nano-encapsulate phytase, which is a thermally unstable biologically active enzyme. Rice bran was converted to nanofibers (20–50 nm in diameter) using electrospinning. After optimizing the pH, viscosity, voltage and the distance between electrodes for electrospinning, phytase enzyme was encapsulated and the fibers were cross-linked using sodium tripolyphosphate. Thermal stability of phytase enzyme was improved by 90 °C when they are encapsulated and cross-linked with sodium tripolyphosphate. The activity of the phytase enzyme was monitored at different temperatures. The activity of the pure enzyme was lost at 80 °C while the enzyme encapsulated into nanofibers demonstrated the activity up to 170 °C. This study opens up many opportunities for nanotechnology value addition to many waste materials and also to improve the properties of a range of biomaterials through a sustainable approach.

## Introduction

Advances in biotechnology have made the industrial-scale production of enzymes possible. Phytase [myo-inositol hexakisphosphate phosphohydrolase], which belongs to the histidine acid phosphatase family is one such enzyme with a high industrial demand. The commercial importance is due to the fact that it initiates the stepwise dephosphorylation of phytic acid, the primary storage form of phosphorus in most seeds and cereal grains, which is about 50–80% of the total phosphorous content in plant tissues [1–3]. The ruminants digest phytic acid with the help of phytase produced by their anaerobic ruminal micro flora. However, monogastric animals such as pig, poultry, and fish are deficient in gastrointestinal tract phytase. Therefore, the addition of phytase into animal feed in many forms to improve the utilization of phosphate from phytic acid has opened up its market volume to over 150 million euro. Hence, phytase has become one of the most widely used enzymes, particularly in poultry (90%) and in swine (79%) industry [4–7].

In recent years, phytase has become important in the human diet as well. For example, a phytase supplemented diet leads to the dephosphorylation of phytase which improves the intestinal uptake of dietary minerals [8, 9].

The problem involved with the phytase use in market products is its thermal instability. The catalytic activity of enzymes depends on the three-dimensional structure of the proteins and the integrity of their native protein conformation. High temperatures can result in substantial changes in these conformations causing the loss of catalytic activity. There are several reports on the denaturation of the phytase enzyme during the palletization process at temperatures around 80 °C [10, 11]. Hence, its industrial applications are substantially challenged. However, enzyme immobilization into a substrate has been used as a suitable technique to achieve this extra stability. For example, immobilization of lipases, re-oxidases, lactases and azo reductases can be given. In addition, such modifications give more convenient handling and more facile separation from the product, thus eliminating the enzyme contamination, efficient recovery and reuse of the costly enzymes enabling the use of continuous, fixed-bed operation [12, 13].

Among the many nanomaterials studied so far, nanofibers (NF) are known as a revolutionary material due to their variety of applications and the extraordinary physical properties they possess, such as greater strength and reactivity [14, 15]. A high surface area to volume ratio of NF, compared to monolithic materials, alone is responsible for these improved physical properties [16]. In the past decade, many scientific advances have been reported in the effective utilization of biopolymers for the synthesis of NF, especially in the field of biomedical engineering [17]. NF has been effectively utilized in controlled drug release, wound dressing, tissue engineering and enzyme immobilization applications [18–23].

We report herein, immobilization of phytase into NF synthesized from rice bran extract using the electrospinning technique, to enhance its thermal stability. Furthermore, rice bran being a natural agricultural byproduct makes this attempt of stabilization of phytase, a sustainable process.

## Materials and methods

### Materials

All the chemicals and reagents used in this study were of analytical grade, purchased from Sigma-Aldrich, USA and used without further purification. Rice bran was obtained from a local rice mill. Phytase enzyme was purchased from Boarding Faithful Industries Co. Ltd, China. The enzyme has been originated from a fungal source *Aspergillus niger.* Poly vinyl alcohol [PVA, MW (60,000–125,000), degree of hydrolysis (98%)] was used to adjust the viscosity of the electrospinning solution.

### Processing of rice bran for fiber synthesis

Rice bran was sieved through a 500 μm mesh screen and was oven dried at 60 °C until a constant weight was obtained. Then, defatting of fibers was done using diethyl ether at a ratio of 1:10 (w/v). Diethyl ether was added to rice bran in a beaker and the system was magnetically stirred for 15 min. Then, the mixture was allowed to settle and the liquid fraction containing fat was carefully removed. This process was repeated two times to ensure the complete defatting and the residue was allowed to stand for few minutes to evaporate any remaining diethyl ether.

Finally, defatted fibers were suspended in water at a ratio of 1:10 (w/v). The pH was adjusted to 9 using 1 mol/dm^3^ NaOH (aq) and heated at 60 °C under stirring for 1 h and allowed to cool to room temperature. Solution pH, conductivity, and viscosity were measured. To separate the base soluble dietary fiber fraction, the solution was centrifuged for 1 h at 8694×*g* of Relative Centrifugal Force. After centrifugation, fiber supernatant was decanted carefully to a container, sealed under a nitrogen environment and kept at 4 °C until further use.

#### Condition optimization for electrospinning

The fiber characteristics resulted from electrospinning depends on pH, solution viscosity, applied voltage and the distance between the anode and the cathode [19]. Therefore, these parameters were optimized accordingly. As solution pH is a very important factor in the process of electrospinning and as well as for the stability of the enzyme, the pH was adjusted subsequently. The viscosity of soluble dietary fiber fraction was not sufficient for electrospinning. Therefore, solutions with various viscosities were made by amending dietary fiber solutions with various PVA percentages (6, 7 and 8%) and the best composition (w/v %) was identified. Since the Taylor cone formation is needed at the end of the needle tip in order to obtain NF, the minimum voltage required to form a Taylor cone was established by increasing the applied voltage from 15 at 1 kV intervals. The distance between two electrodes was set to 10, 15 and 20 cm and fibers were made accordingly. Fiber morphology was observed with the use of scanning electron microscope. The best distance to obtain a uniform, nanosized diameter fibers was decided.

#### Synthesis of NF using electrospinning

The supernatant containing soluble fibers were used to electrospinning of NF. For this, fiber supernatant was amended with PVA at a concentration of 8% (w/v) to gain the required viscosity. The temperature was increased to 100 °C to completely dissolve PVA under stirring until a homogeneous solution was obtained. Then, the solution was left to cool down to 30 °C. The viscosity and pH of the solution were measured. The solution was filled to a syringe and fixed to the electrospinner (home engineered). The ground electrode (collector plate) was covered with an aluminum foil and electrospinning parameters were set as follows: tip collector distance, 15 cm, and accelerating voltage 27 kV. The flow rate was set manually until a Taylor cone was observed. After electrospinning, fibers were collected carefully and packed under a vacuum.

#### Synthesis of cross-linked surface modified nanofibers with phytase enzyme (CSNFP)

About 350 mg of NF were dipped in a phytase enzyme solution (112.29 unit/mL) and kept in a shaker (100 rpm) for 1 h. Then, the excess enzyme was drained off. In order to cross-link, sodium tripolyphosphate (STTP) solution (1.5 w/v %) was added to the fibers, drop wise for 5 min. The excess STPP was washed off thoroughly by dipping in three deionized water baths for 1 min in each. The resulted fibers were dried in a vacuum oven for 12 h at 25 °C and stored at 4 °C.

### Characterization of NF and CSNFP

Thermal profiles of nanofiber composites were obtained under a nitrogen atmosphere (100 mL/min) using TA Instruments SDTQ600. The sample (5–15 mg) was filled into an alumina pan and heated from ambient temperature to 1000 °C at a rate of 10 °C/min. Data were acquired by TA Q series, version 5.4.0 and analyzed using universal analysis 2000, version 4.5. Melting temperatures (T_m_) and melting enthalpies (ΔH_m_) were determined using TA Instruments SDTQ200 under nitrogen flow rate of 50 mL/min. Samples (5–10 mg) were heated from 0 to 400 °C at a rate of 10 °C/min in a t-zero aluminum pan. Data were acquired by TA Q series, version 5.4.0 and the results were analyzed using universal analysis 2000, version 4.5.

The fiber size and the morphology of samples were analyzed using HITACHI SU6600 Scanning Electron Microscope. The sample was mounted onto a double-sided carbon tape on an aluminium stub and sputtered with gold using HITACHI, E-1020 Ion Sputter. The SEM images were recorded at 20 kV using the secondary electron detector.

UV–Vis spectrophotometric analysis was done using UV–Vis–NIR spectrophotometer (UV 3600, Shimadzu, Japan) in the absorbance mode. Absorption spectra were analyzed using UV-Prob 2.33.

### Determination of the activity of CSNFP

Synthesized CSNFP (5.0 mg) was weighed to a storage vial and kept in a water bath at 37 °C for 15 min. Phytic acid solution (0.5478 mmol/dm^3^) prepared using glycine buffer (pH 2.5) was kept in a water bath at 37 °C for 15 min. Phytic acid solution (15 mL of 0.5478 mmol/dm^3^) was mixed with CSNFP and incubated at 37 °C for 20 min. The reaction was terminated by adding 12.5 mL of vanadomolybdate reagent. The amount of inorganic phosphate resulted from the enzymatic reaction was determined using the standard vanadomolybdate UV spectrophotometric method [24].

### Determination of the stability of CSNFP under different temperatures

The synthesized CSNFP (5.0 mg) and an equal amount of free enzyme solutions were heated starting from 50 to 170 °C at 10 °C intervals, and kept in a water bath (up to 100 °C) and in a mineral oil bath (when the temperature above 100 °C) respectively, for 5 min. After 5 min, the samples were cooled down to room temperature and the activity was measured [24].

## Results and discussion

Pretreatment of the fiber material is really important to obtain good quality fibers. Dried and sieved rice bran should be defatted to remove the fat content (17%) [25] which could lead to the formation of emulsions during the electrospinning process. Emulsion formation can affect the physical parameters of the polymer solution as well as the fiber morphology. The defatted rice bran is then subjected to an alkali treatment to extract soluble dietary fibers (SDF) (pectin, proteins, carbohydrate and some amount of cellulose) [19]. The solid content of the supernatant was 30%.

After the extraction of SDF, the condition optimization for the electrospinning was done in order to avoid the formation of film and beads-on-string structures. Electrospun fibers with these artifacts are usually considered as “poor” quality fibers since they can reduce the total surface area significantly. As a result, these structures can directly reduce the enzyme loading capacity of the fibers thus leading to poor industrial sustainability.

### Condition optimization

#### Solution pH

High conductivity or charge density results in more uniform fibers with fewer beads-on-string structures [26]. However, in this study, both extremes of pH values are not desirable since the enzyme undergoes denaturation at high pH values while the PVA molecules used as the viscosity modifier can undergo protonation at lower pH values leading for the formation of beads-on-string structures instead of uniform NF [26]. The optimum solution pH for electrospinning was set at 9.

#### Solution viscosity

Solution with 6% (w/v) PVA results in droplets and beads-on-structures rather than drawing into fibers. Smooth fibers with a very small amount of beads resulted when electrospinning was done with the dietary fiber supernatant amended with 8% (w/v) PVA. The solution viscosity and conductivity were optimized as 800 Cp, and 17.0 mS/cm, respectively.

#### Applied voltage

Applied voltage is another important parameter in determining the fiber morphology. The mechanism of charge transfer from the tip/needle to the collector through the flow of polymer is due to the voltage difference between two electrodes. Therefore, an increase in electrospinning current coincides with the mass flow rate away from the tip [25] as the applied voltage is proportional to the fiber diameter. In this study, the best quality nanosized fibers were obtained at a minimum applied voltage of 27 kV.

#### The distance between two electrodes

The distance between the needle and the collector is another parameter that determines the morphology and the diameter of the electrospun fibers. The distance has to be maintained in such a way that the fibers would get sufficient time to dry, before reaching the collector, by evaporation of the solvent used in fiber solution. Distances that are too close produced bead-on-fiber structures. When the distance was too long, the fibers started to gather at the edges of the collector. Droplet formation was observed at 10 cm distance, while the very small amount of fibers were formed at the edge of the collector at 20 cm. At 15 cm, a reasonable amount of fibers with nanoscale diameters were collected at the electrode. Hence, the distance between the needle and the collector was set as 15 cm.

### Morphology of synthesized NF and CSNFP

**Fig. 1:**
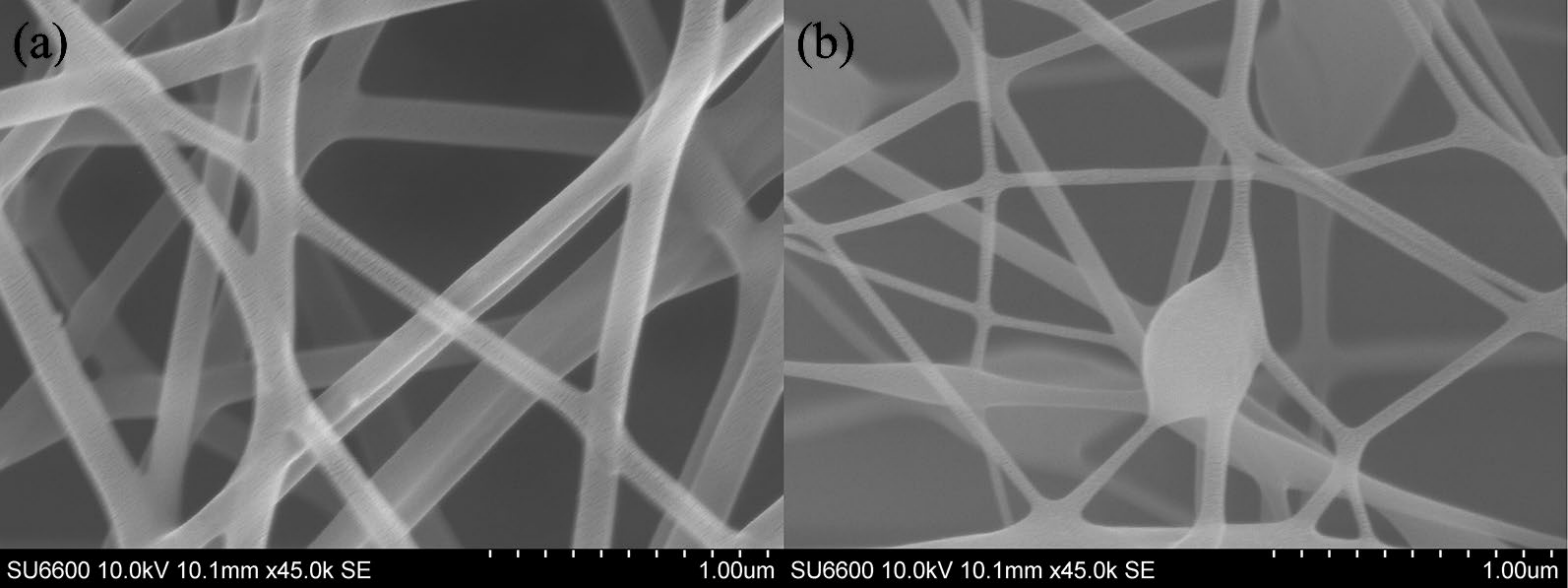
Scanning electron microscopic images of synthesized nanofiber composites **a** NF, and **b** CSNFP

The dietary fiber solution was successfully spun into NF and obtained a network structure with fiber diameter between 30 and 50 nm (Fig. 1a).

The fiber surface was smooth and a minimum number of beads-on-string structures were observed. These fibers were less than half the size of those reported, previously [19]. Interestingly, less morphological changes can be seen after surface modification of NF with phytase enzyme in the presence of STTP. However, a large number of beads of 400 nm diameter have been observed on the fibers of CSNFP composites. This network structure can offer an extra stability to the enzyme since it gives a better protection from external conditions. Also, the successive addition of negatively charged STTP might lead to the formation of crosslinks with positively charged ammonium groups in CSNFP. Moreover, NF might offer a high surface area for efficient encapsulation of the enzyme.

### Thermal properties of synthesized NF

**Fig. 2:**
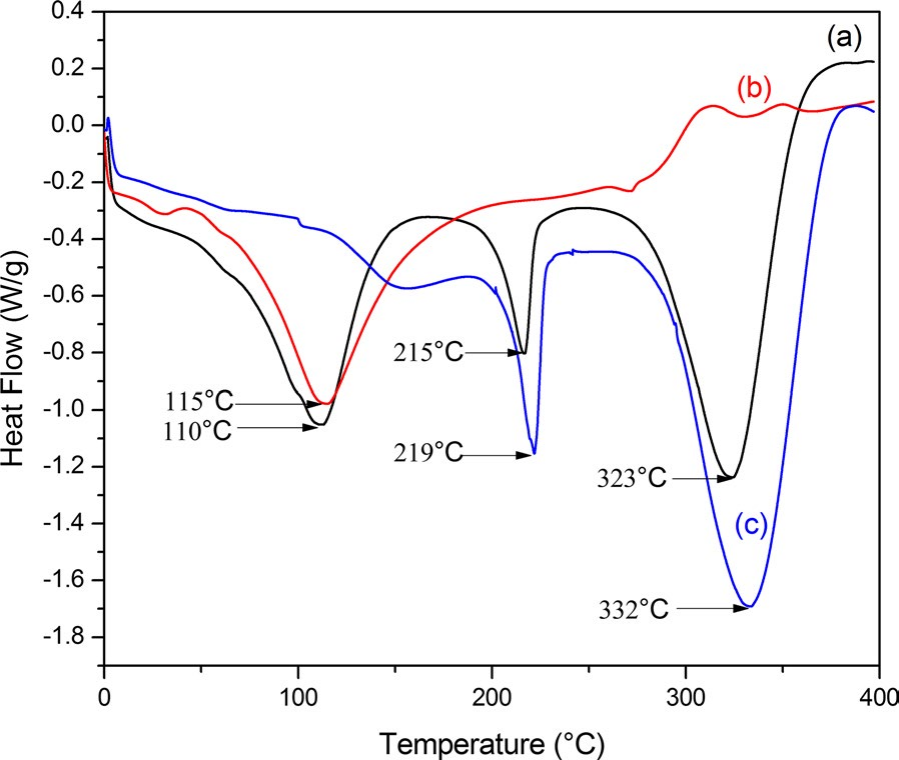
DSC thermogram of (a) NF, (b) rice bran, and (c) PVA

Differential scanning calorimetry (DSC) thermogram of the synthesized NF basically showed three main endotherms (Fig. 2).

The characteristic pattern is very similar to those reported by Zhang et al. [27] on PVA-soy blend NF and Fung et al. [19] on agro waste such as soya bean solid wastes, oil palm trunk and oil palm frond based NF. The first endotherm centered at 110 °C corresponds to the melting of the total SDF and other organic components present in the composite. The second endotherm centered at 215 °C can be attributable to the melting of PVA component, while the endotherm at 323 °C is related to subsequent decomposition of the composite under nitrogen environment.

**Fig. 3:**
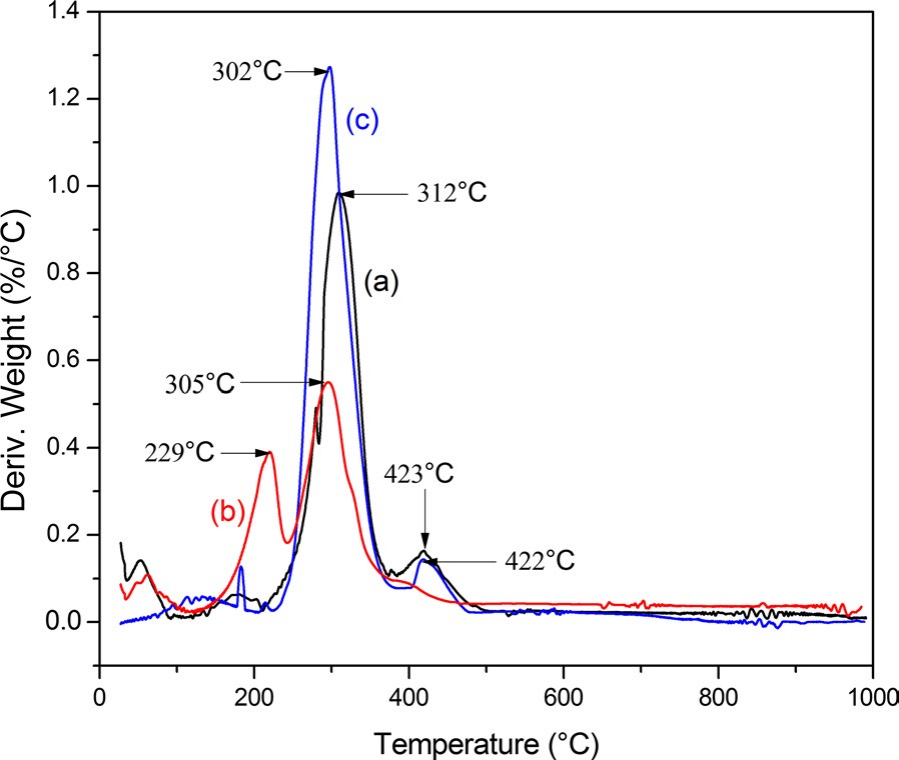
DTA thermogram of (a) NF, (b) rice bran, and (c) PVA

First order differential thermal analysis (DTA) thermogram of NF is given in Fig. 3 compared to both individual PVA and rice bran.

For NF, weight losses were observed in three different temperature ranges (i) 39–99 °C, accounting for the removal of physisorbed water, (ii) 100–312 °C due to the loss of acetyl groups by transforming to acetic acid molecules with subsequent in situ chain stripping from partially acetylated PVA [19]. Also, the same phenomenon can be expected from pectin substances present in SDF (iii) 312–425 °C, chain scission and pyrolysis of PVA and SDF. Comparing the relevant values for both individual PVA (302 °C) and rice bran (230 °C) the thermal decomposition value of NF has been shifted to 312 °C indicating an extra thermal stability in the composite.

### Thermal properties of synthesized CSNFP

**Fig. 4:**
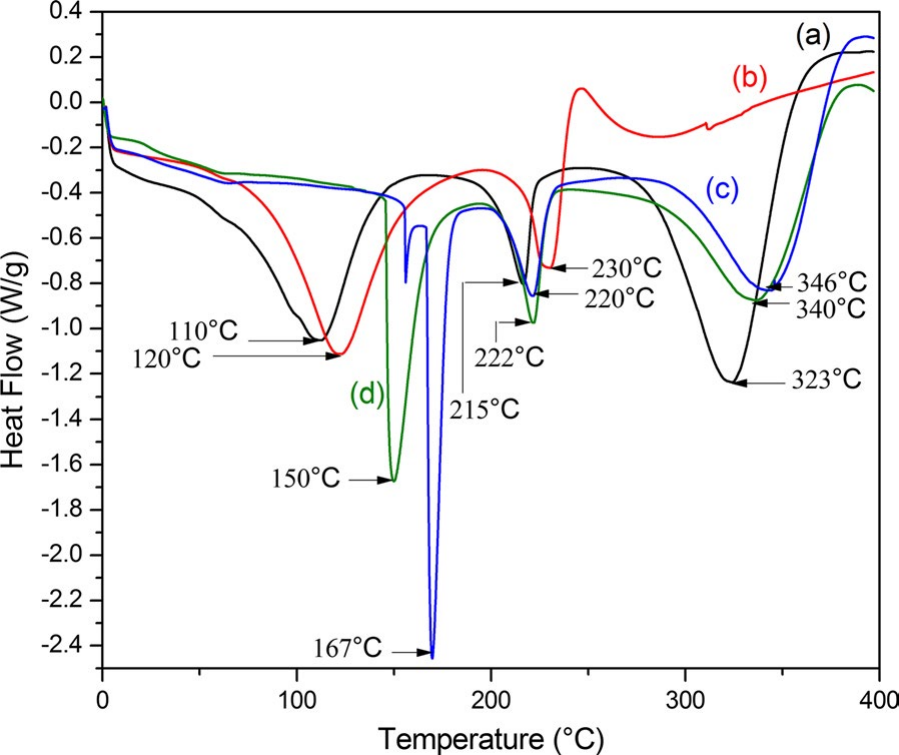
DSC of (a) NF, (b) phytase enzyme, (c) CNF, and (d) CSNFP

CSNFP exhibited endotherms at 150 and 222 °C which are due to the melting of SDF and PVA components in the composite and at 340 °C which is due to the subsequent decomposition (Fig. 4).

As can be seen in Fig. 4, the endotherm corresponding to the melting of the SDF at 110 °C in NF has been shifted to a higher temperature (150 °C) in CSNFP, indicating a better interaction between pectin, cellulose and other biopolymers and stronger interaction in between NF due to the cross-linking effect. This can be clearly seen in cross-linked NF (CNF) which is working as the control experiment for CSNFP.

Similarly, the melting corresponding to the PVA component has been shifted into a higher value in CSNFP (222 °C) and CNF (220 °C) compared to NF (215 °C). This is due to the same reason explained above. Decomposition temperatures of the fibers also have increased accordingly from 323 to 340 and 346 °C.

The melting point of phytase enzyme also has been shifted from 120 to 150 °C in CSNFP, which can be considered as a significant increase of the physical thermal stability of the enzyme. This could be again due to the strong interactions between the enzyme molecules and functional groups in NF and also due to cross-links in between enzyme molecules in the composite. First mentioned interactions could be assigned as strong hydrogen bonds between surface –OH groups in NF and –NH groups and –C=O groups in the enzyme molecules.

**Fig. 5:**
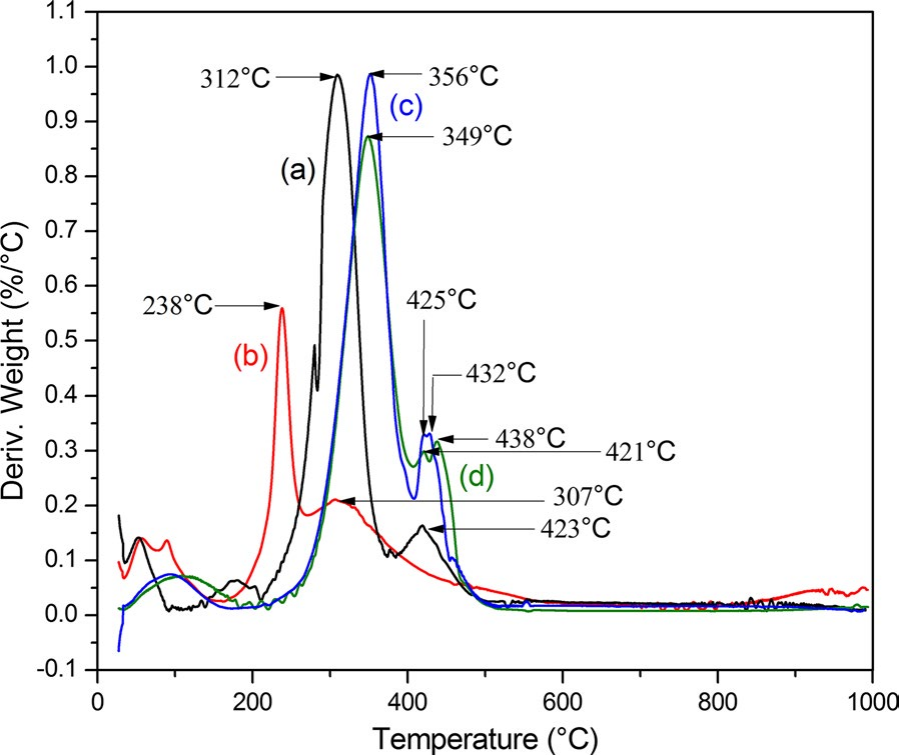
DTA of (a) NF, (b) phytase enzyme, (c) CNF, and (d) CSNFP

DTA data indicates two regions of maximum weight losses in NF at 312 and 423 °C, while in CSNFP there are three regions at 349, 421 and 438 °C (Fig. 5).

These can be attributed to the corresponding thermal decomposition. The thermal decomposition step at 312 °C in NF has been shifted to 348 °C in CSNFP. This is due to the thermal stability attained by the cross-linking of fibers with STPP. Due to the cross-linking, fiber strands are held together strongly making it resistant to thermal decomposition. This observation is further assisted by the decomposition pattern of CNF. It shows three decomposition steps of CNF at 356, 425 and 432 °C are higher than the values of NF which can be attributed to the proof of concept.

The thermal decomposition step at 238 °C in phytase enzyme cannot be seen in the CSNFP. It has been shifted to 348 °C, which is a significant thermal stabilization of the enzyme. This could be due to the strong hydrogen bonds between surface hydroxyl (OH) groups in NF and amine (NH) groups and carbonyl (C=O) groups in the enzyme molecules. Cross-links between enzyme molecules–enzyme molecules, enzyme molecules–NF, and NF–NF through STPP might have also affected the thermal stability of the enzymes.

### Determination of the activity along with thermal stability of CSNFP

The activity of CSNFP was found as 0.1 units/mg. An extra thermal stability of the enzyme was expected due to the multi-point or multi-subunit immobilization to the fiber surface [28].

**Fig. 6:**
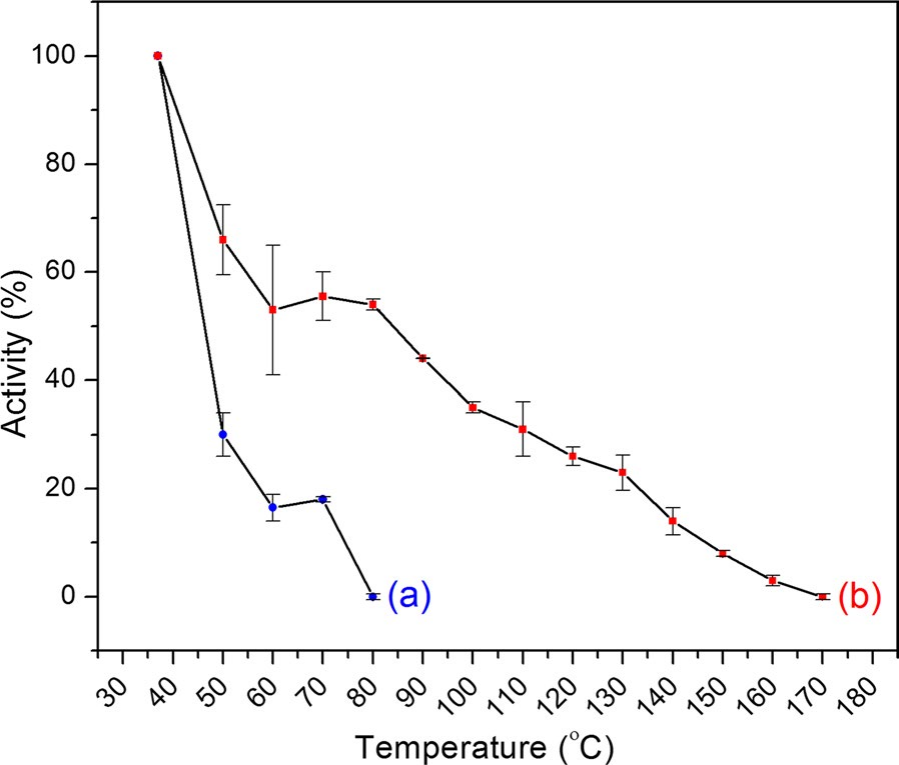
Activity (%) of (a) free phytase enzyme and (b) CSNFP, after being exposed to different temperatures

For phytase enzyme, the optimum working temperature is around 40–45 °C [29]. As the temperature continues to rise above the optimum, the activity and the rate of the reaction decrease abruptly due to the denaturation of enzyme structure. In this study, we observed that for free phytase enzyme the activity was entirely lost around 70–80 °C (Fig. 6).

This observation compares well with the value reported by Wyss et al. [30], as they have observed a complete denaturation of the phytase enzyme extracted from *A. niger* at temperatures between 50 and 70 °C [30]. Further, it has been reported that the denaturation takes place during the palletization process at temperatures around 70–80 °C [10, 11], which was also evidential in our study.

In contrast to free enzyme, the CSNFP have shown a significant improvement in the thermal stability as it continued to show the enzymatic activity up to 170 °C, an improvement by 100 °C.

Generally, there are two processes responsible for the activity loss of an enzyme with temperature. First, denaturation which usually happens because of the loss of tertiary (and often secondary) protein structure which is not involved in covalent bond cleavage which is theoretically reversible. Second, the degradation which is the loss of primary structure, associated with covalent bond cleavage and/or formation, which is irreversible [31]. Either one or both can account for the inactivation of the enzyme at elevated temperatures, usually above 80 °C. The upper-temperature limit for enzyme stability depends on the conformational integrity of the enzyme or protein. Greater the conformational integrity, greater is the stability. Greater thermal stability of the surface modified enzyme carrying NF may be due to the immobilization of enzymes on the nanofiber surface. Strong hydrogen bonding can be expected between the surface –OH groups present in synthesized NF and free –C=O and –NH groups present in the phytase enzyme. PVA and the dietary fibers are responsible for the surface –OH groups in the NF. Subsequent cross-linking with STTP may have further increased the thermal stability. Strong hydrogen bonding between –O–P groups in STTP and free –NH groups in enzyme and –OH groups in the nanofiber surface a responsible for stable cross-links between enzyme/enzyme, enzyme/NF, and NF/NF.

## Conclusions

It can be concluded that the rice bran is a suitable candidate to synthesize dietary fibers in nanoscale using electrospinning technique. In this study, a thermally unstable enzyme phytase had been successfully incorporated into these nanofibers. The enzyme incorporated fibers demonstrated improved thermal properties in which the enzyme denaturation temperature had increased from 80 to 170 °C. Therefore, the findings open up new pathways for stabilization of bio-molecules in nanofibers based on agriculture waste materials.

## Abbreviations

NF: nanofibers
PVA: poly vinyl alcohol
CNF: cross-linked nanofibers
CSNFP: cross-linked surface modified nanofibers
STTP: sodium tripolyphosphate
SDF: soluble dietary fibers
DSC: differential scanning calorimetry
DTA: differential thermal analysis
